# Predictive effect of the triglyceride glucose index on abnormal blood glucose metabolism events in populations with sarcopenia: a cross-sectional study

**DOI:** 10.1186/s12902-025-02026-8

**Published:** 2025-09-30

**Authors:** Yingying Zhu, Jiabei He, Xin Li, Yunping Xu, Jingshuang He, Liang Li

**Affiliations:** 1 Department of Geriatric Medicine, Zhongshan Hospital of Traditional Chinese Medicine, Zhongshan, Zhong Shan, Guangdong Province 528400 China; 2 Department of Preventive healthcare, Zhongshan Hospital of Traditional Chinese Medicine, Zhongshan, Zhong Shan, Guangdong Province 528400 China

**Keywords:** Triglyceride-Glucose index, Sarcopenia, Abnormal blood glucose metabolism events, Muscle mass reduction, Cross-sectional study

## Abstract

**Background:**

Recent studies reveal that obesity and reduced muscle mass are key factors implicated in insulin resistance (IR) and abnormal glycemic metabolism. Nonetheless, it remains ambiguous which demographic, particularly those with obesity or diminished muscle mass, exhibits a greater vulnerability to insulin resistance.

**Objective:**

The aim of this study is to investigate the relationship between insulin resistance assessed by the triglyceride and glucose index (TyG index) and abnormal blood glucose metabolism in individuals with both reduced muscle mass and obesity. Furthermore, we assess the efficacy of the TyG index in forecasting occurrences of atypical blood glucose metabolism within this particular demographic.

**Methods:**

This cross-sectional study utilized data collected from adults who underwent a health assessment at our institution between December 2022 and September 2024. The evaluation of insulin sensitivity was conducted utilizing the TyG index, whereas muscle mass was quantified through bioelectrical impedance analysis (BIA). Logistic regression, Receiver Operating Characteristic (ROC) curve analysis, subgroup analysis, and interaction effect analysis were employed to evaluate the association between triglyceride-glucose index levels with subgroups based on different body masses.

**Results:**

This study comprised 1278 people with diminished muscle mass. We found a positive association between TyG index (OR = 7.73; 95% CI: 4.62–13.51; *P* < 0.001) and the risk of abnormal glucose metabolism among those who were overweight/obese. The analysis of the ROC curve revealed that the TyG index possesses a significant predictive capability for identifying abnormal glucose metabolism in individuals exhibiting diminished muscle mass (AUC = 0.906). This predictive value is particularly pronounced in those who do not present with overweight or obesity (AUC = 0.915), in contrast to its effectiveness in individuals who have both reduced muscle mass and overweight or obesity. Subgroup analyses confirmed the positive association of TyG index with abnormal blood glucose incidence.

**Conclusion:**

This study assessed the link between TyG and abnormal blood glucose metabolism events risk in people with sarcopenia and compared its predictive power for abnormal blood glucose metabolism events onset in those with low muscle mass but not overweight or obese. We recommend using the TyG index as the best predictive marker for assessing abnormal blood glucose metabolism events risk in individuals without concurrent overweight or obesity, regardless of their body mass index.

**Supplementary Information:**

The online version contains supplementary material available at 10.1186/s12902-025-02026-8.

## Introduction

Type 2 diabetes (T2DM) arises from complex genetic-environmental interactions, progressing from insulin resistance (IR) to β-cell dysfunction [1]. Sarcopenia, defined by declining muscle mass, strength, and physical performance [2], shares a bidirectional relationship with T2DM. Skeletal muscle—the major site of glucose metabolism—atrophies with age, exacerbating IR and dysglycemia [3]. Conversely, IR impairs muscle protein synthesis, accelerating sarcopenia [4, 5]. This synergy is particularly pronounced in sarcopenic obesity (SO), where chronic inflammation and metabolic dysfunction amplify both conditions [6–8].

The triglyceride-glucose (TyG) index, a validated IR surrogate [9–11], outperforms HOMA-IR in correlating with hyperinsulinemic-euglycemic clamp results [12–15]. Though widely used in metabolic studies, its utility in populations with concurrent muscle loss and dysglycemia remains underexplored. The TyG index has shown commendable efficacy in precisely detecting insulin resistance among various populations [16–18]. The TyG index outperforms HOMA-IR and demonstrates broad applicability in epidemiological research.

While many studies have indicated a link between the decline in muscle mass and insulin resistance, irregular glucose metabolism, and diabetes, most investigations regarding the utilization of the TyG index have centered on the general population. Limited investigations have explored the use of the TyG index to predict abnormal glucose metabolism events in individuals with muscle mass loss. Therefore, this study aims to retrospectively analyze health examination data using the TyG index to evaluate its potential correlation with abnormal glucose metabolism events in individuals experiencing both muscle mass loss and overweight/obesity.

## Materials and methods

### Study population

**Fig. 1:**
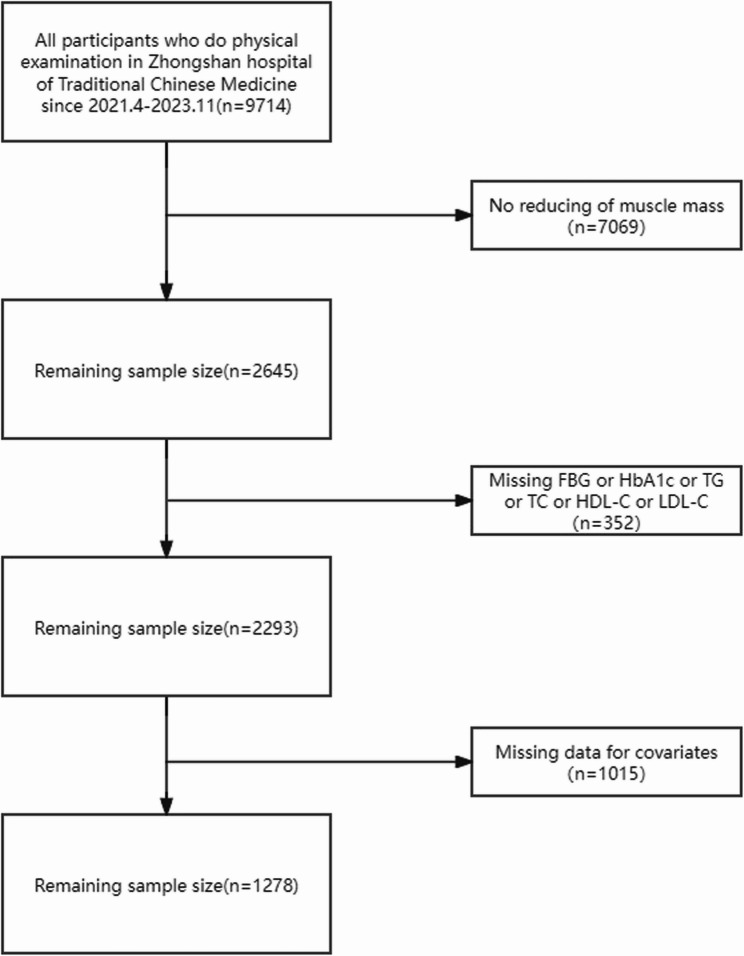
Flowchart

A total of 9,714 participants were enrolled from December 2022 to September 2024.The inclusion criteria for participants are individuals aged 18 years or older who are Han Chinese and are undergoing health check-ups at our hospital (both male and female). Bioelectrical impedance analysis (BIA) was employed to assess individual muscle mass, and participants with normal muscle mass were excluded from this study.We eliminated individuals from the analysis who had incomplete data for Triglyceride (TG), Total Cholesterol (TC), Fasting Serum Glucose (FSG), blood pressure, Glycated Hemoglobin A1C(HbA1c), High-Density Lipoprotein (HDL-C), Low Density Lipoprotein (LDL-C) or initial muscle mass reduction. As a result of this exclusion criteria, our final sample consisted of 1278 participants **(**Fig. 1**)**. The study was approved by the Ethics Committee of Zhongshan Hospital, Guangzhou University of Chinese Medicine and adhered to the principles outlined in the Declaration of Helsinki. In this cross-sectional study, written informed consent was not procured, given that all data were anonymized.

### Definition of abnormal blood glucose metabolism events [19]

Diabetes was defined as having FSG ≥ 126 mg/dl, HbA1c ≥ 6.5%, previous diagnosis of new-onset abnormal blood glucose metabolism events, or use of antidiabetic medications. Prediabetes was defined as FSG of 100 mg/dL (5.6 mmol/L) to 125 mg/dL (6.9 mmol/L) Impaired Fasting Glucose(IFG) or HbA1c of 5.7–6.4% (39–47 mmol/L) or 2 h plasma glucose(2-h PG) during 75-g Oral Glucose Tolerance Test(OGTT) 140 mg/dL (7.8 mmol/L) to 199 mg/dL (11.0 mmol/L) Impaired Glucose Tolerance(IGT). Normal fasting glucose was defined as without diabetes or prediabetes. Abnormal blood glucose metabolism events were defined diabetes and prediabetes.

### Definition of muscle mass reduction [20]

In accordance with the criteria outlined by the “Asian Working Group for Sarcopenia: 2019 Consensus Update on Sarcopenia Diagnosis and Treatment” for diagnosis:

$$\begin{aligned}&\text{Bioelectrical impedance analysis (BIA)}\!: \\&\text{Men}<7.0 \text{kg/m}^{2}, \text{Women}<5.7 \text{kg/m}^{2}\end{aligned}$$

This study primarily employed the Bioelectrical Impedance Analysis (BIA) method to assess muscle mass. The measurements were conducted using a body composition analyzer, specifically the InBody 270 model, manufactured by InBody Corporation in South Korea.

### Assessment of TyG index and BMI

In this study, fasting glucose and triglyceride levels were measured after an 8.5-h fasting period using an automated biochemical analyzer.

$$\text{TyG}=\text{ln}\;[\text{TG}\;(\text{mg/dl})\times\text{FSG}\;(\text{mg/dl})/2]$$

The body mass index (BMI) (kg/m^2^) was derived by dividing the weight (kg) of the individual by the square of their height (m).

### Definition of overweight and obesity [21–23]

#### Overweight and obesity

A BMI of 24 kg/m^2^ or more is considered overweight and obesity.

### Covariate adjustment

We utilized covariate adjustment models to evaluate the impact of confounding variables on the relationship between TyG and the reduction of muscle mass in individuals with abnormal blood glucose metabolism events. The covariates considered were age (in years), sex (male/female), diastolic blood pressure, uric acid levels, and creatinine levels.

### Statistical analysis

Continuous variables were presented as mean (standard deviation, SD) for normal distributions and as median (interquartile range, IQR) for skewed distributions. Dichotomous variables were articulated in terms of frequency and percentage. The t-test was used for continuous variables, while Pearson’s chi-square test was employed for categorical variables. The baseline characteristics’ categorical variable proportions (%) were compared using the chi-square test. A multivariate logistic regression model assessed the odds ratio (OR) and 95% confidence interval (CI) to determine the association between TyG and abnormal blood glucose metabolism events in muscle mass reduction. The joint probability was computed, correlating muscle mass reduction with obesity; a ROC curve was generated to ascertain the area under the curve (AUC), and then, the Jordan index identified the best cut-off value. A P-value < 0.05 indicated statistical significance. To address the issue of mixed variables, we constructed three multivariable adjustment models. Model 1 does not adjust for covariates; Model 2 adjusts for age and sex. Model 3 incorporates additional adjustments for systolic blood pressure, diastolic blood pressure, uric acid level, and creatinine level based on Model 2.

To clarify the statistical methodology and interpretation of interaction analyses, we employed a comprehensive approach to explore the potential effect modifications in the association between the TyG index and diabetes onset across different subgroups. Interaction analyses were performed to evaluate whether the relationship between TyG index and diabetes varied according to stratified factors, including sex, age, hypertension, hyperuricemia, and creatinine clearance levels. Specifically, interaction terms were added to the logistic regression model to assess statistical significance.

The statistical rationale for interaction analyses stems from the need to identify effect heterogeneity, which provides a deeper understanding of whether and how certain subgroups respond differently to the exposure of interest (TyG index). The interaction effects were quantified using P-values for interaction terms, with a significance threshold set at *P* < 0.05.

All statistical analyses were conducted using R version 4.3.2 software (https://www.r-project.org/). In the regression analysis, variables with P-values < 0.10 in univariate and multivariate regression analyses were included, and results are reported as beta coefficients with corresponding 95% confidence intervals (OR: odds ratio; CI: confidence interval). A significance threshold of *P* < 0.05 was applied.

## Result

### Baseline characteristics of study participants

Baseline Characteristics Table among the 1278 participants included in this study, there were 503(39.36%) males and 775(60.64%) females, with a mean age of 36.80 ± 10.70 years. Out of these, 62(4.9%) were classified as overweight/obese while the majority, i.e.,1216(95.1%), were not overweight/obese individuals. The analysis indicated that a reduction in muscle mass was noted in overweight and obese women by approximately 20.97%, while in men, the reduction was about 79.03%. In contrast, muscle mass reduction without being overweight/obese accounted for around 62.6% among women and 37.4% among men, respectively. The two groups showed significant differences in terms of sex ratio, age, blood pressure levels, fasting blood glucose levels, blood lipid profiles, uric acid levels, and skeletal muscle index as well as TyG index values (*P* < 0.05). Compared to non-overweight/obese individuals, the overweight/obese group exhibited older age and higher likelihood of hypertension occurrence along with metabolic abnormalities related to blood glucose levels, blood lipid profiles, and uric acid metabolism (Table 1).

**Table 1:** Baseline clinical characteristics of participants with reduced muscle mass by BMI status

	Total (*n* = 1278)	BMI < 24 kg/m^2^ (*n* = 1216)	BMI ≥ 24 kg/m^2^ (*n* = 62)	*P*-value
Sex				< 0.001
Male, *n*(%)	503 (39.36%)	454 (37.33%)	49(79.03%)	
Female, *n*(%)	775 (60.64%)	762 (62.66%)	13 (20.97%)	
Age(Years)	36.80 ± 10.70	36.49 ± 10.59	42.90 ± 11.10	< 0.001
Height(m)	1.60 ± 0.07	1.60 ± 0.07	1.61 ± 0.07	0.074
Weight(kg)	51.18 ± 7.42	50.45 ± 6.66	65.54 ± 6.80	< 0.001
BMI(kg/m2)	20.02 ± 2.29	19.76 ± 1.98	25.21 ± 1.71	< 0.001
SBP(mmHg)	114.07 ± 12.93	113.34 ± 12.36	128.50 ± 15.29	< 0.001
DBP(mmHg)	69.37 ± 9.88	68.76 ± 9.30	81.32 ± 12.94	< 0.001
Hypertension				< 0.001
Yes	68(5.32%)	51 (4.19%)	17(27.42%)	
No	1210(94.68%)	1165(95.81%)	45(72.58%)	
FBG(mg/dL)	93.21 ± 29.96	92.51 ± 29.12	107.06 ± 41.13	< 0.001
Diabetes				< 0.001
Yes	67(5.24%)	54(4.44%)	13(20.97%)	
No	1211(94.76%)	1162(95.56%)	49(79.03%)	
TyG index	8.35 ± 0.60	8.32 ± 0.56	9.01 ± 0.89	< 0.001
UA(umol/L)	339.20 ± 93.42	334.91 ± 91.18	423.45 ± 97.65	< 0.001
CR(umol/L)	60.28 ± 14.84	59.81 ± 14.77	69.45 ± 13.14	< 0.001
LDL(mmol/L)	2.93 ± 0.86	2.91 ± 0.84	3.35 ± 1.09	< 0.001
HDL(mmol/L)	1.49 ± 0.38	1.50 ± 0.38	1.28 ± 0.34	< 0.001
TG(mmol/L)	1.25 ± 1.22	1.18 ± 0.99	2.59 ± 3.14	< 0.001
TC(mmol/L)	5.02 ± 0.97	4.99 ± 0.95	5.56 ± 1.08	< 0.001

Data are reported as no.(%)or mean ± standard deviations,*P*-value were derived from χ2 test or Student’s test,*P*- value<0.05,*BMI *Body Mass Index, *TyG* Triglyceride Glucose index, *SBP* Systolic Blood Pressure, *DBP* Diastolic Blood Pressure, *FBG* Fasting Blood Glucose, *UA* Uric acid, *CR* Serum Creatinine level, *LDL* Low Density Lipoprotein, *HDL* High-Density Lipoprotein, *TG* Triglyceride, *TC* Total Cholesterol.

### Multiple logistic regression analysis of the factors affecting abnormal blood glucose metabolism events in patients with different types of muscle mass reduction

A comprehensive analysis employing both univariate and multivariate regression techniques was undertaken, utilizing abnormal blood glucose metabolism events as the dependent variable. Variables exhibiting a significance level of *P* < 0.1 were meticulously screened for inclusion in the equation. Due to multicollinearity concerns between fasting blood glucose levels, high-density lipoprotein levels, triglyceride levels, and TyG, these variables were excluded from consideration. Ultimately, sex, age, diastolic blood pressure level, serum creatinine level, and skeletal muscle index were selected as independent variables. The multivariate logistic regression analysis conducted on all patients with sarcopenia, independent of their comorbid overweight or obesity status, demonstrated a significant correlation between TyG and the onset of abnormal blood glucose metabolism events (*P* < 0.05) **(**Table 2**)**.

**Table 2:** Multivariate logistic regression between TyG index and abnormal blood glucose metabolism events in populations with different types of sarcopenia

Characteristics	Odds Ratio(95%CI)	*P*-value	VIF
Total	7.73(4.62–13.5)	< 0.01	1.2
BMI < 24 kg/m^2^	9.86(5.67–18.3)	< 0.01	1.2
BMI ≥ 24 kg/m^2^	3.42(1.19–16.10)	0.021	1.6

Data are presented as odds ratios, 95% confidence intervals, and *P*-value

*OR* Odds Ratio, *CI* Confidence Interval, *TyG* Triglyceride Glucose index,*VIF* Variance Inflation Factor

### Covariate-adjusted model

In order to assess the robustness of our model, we performed covariate adjustments in two models. Model 1 incorporated modifications for age and sex, whereas model 2 further accounted for diastolic BP level, uric acid level, and serum creatinine level. Subsequent to the modifications, a significant relationship became apparent between the decrease in total muscle mass and the specified variables (*P* < 0.05). While Model 2 showed consistent TyG-dysglycemia associations overall (OR = 7.82, *P* < 0.001), the relationship was attenuated in the BMI ≥ 24 subgroup (OR = 2.98, *P* = 0.08), possibly due to competing metabolic effects of adiposity **(**Table 3).

**Table 3:** Covariate adjustment models between TyG index and abnormal blood glucose metabolism events in populations with different types of reduced muscle mass

Characteristic	Crude model OR(95%CI)	*P*- value	Model 1 OR(95%CI)	*P*-value	Model 2 OR(95%CI)	*P*-value
Total	10.56(6.82–16.34)	*P* < 0.001	8.83(5.42–14.39)	*P* < 0.001	7.82(4.60-13.29)	*P* < 0.001
BMI < 24 kg/m^2^	13.55(8.08–22.73)	*P* < 0.001	11.43(6.50-20.09)	*P* < 0.001	11.03(5.90-20.61)	*P* < 0.001
BMI ≥ 24 kg/m^2^	2.88(1.30–6.3)	*P* < 0.001	2.73(1.09–6.86)	*P* = 0.003	2.98(0.87–10.21)	*P* = 0.08

Data are presented as odds ratios, 95%confidence intervals, and *P*-value

Crude model adjusts for None

Model 1 adjust for Age (years), Sex (male/female)

Model 2 adjust for Age (years), Sex (male/female), Diastolic blood pressure level, Uric acid level, Serum creatinine level

*OR* Odds Ratio, *CI* Confidence intervals, *TyG* triglyceride glucose index

###  TyG index and the predictive value of abnormal blood glucose metabolism events in all muscle mass reduction populations

**Fig. 2:**
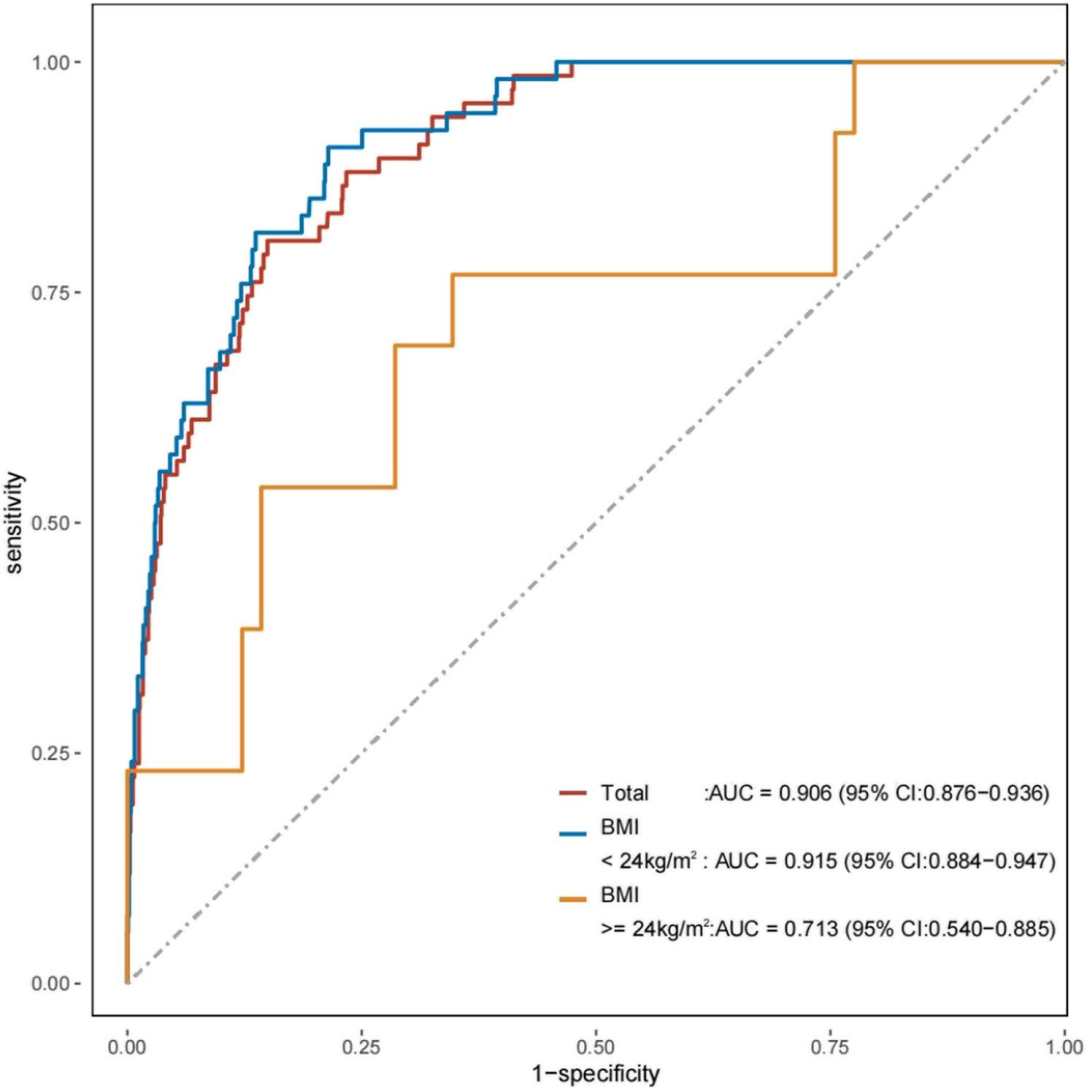
ROC curves for each index as predictors of abnormal blood glucose metabolism events

Figure 2 illustrates the ROC curves corresponding to various indicators. The values for sensitivity, specificity, and Youden’s index are delineated in Table 4. In the examination of ROC curves, within the cohort experiencing muscle mass reduction, TyG revealed an area under the curve (AUC) of 0.906 for the diagnosis of type 2 abnormal blood glucose metabolism events, indicating a sensitivity of 0.806 and a specificity of 0.851;ROC curve analysis identified 0.877 as the optimal cut-off value (maximizing sensitivity and specificity); the Youden Index was 0.66. Within the subgroup without coexisting overweight/obesity but with reduced muscle mass, TyG displayed an AUC of 0.915 for diagnosing abnormal blood glucose metabolism events, accompanied by a sensitivity of 0.907 and a specificity of 0.786; ROC curve analysis identified 8.57 as the optimal cut-off value (maximizing sensitivity and specificity); the Youden Index was 0.40.In contrast to this subgroup, within the group characterized by both reduced muscle mass and coexisting overweight/obesity, the AUC for TyG in diagnosing abnormal blood glucose metabolism events was found to be lower at only0 0.713,with a sensitivityofonly7.70 and a specificity of only 6.50;ROC curve analysis identified 8.88 as the optimal cut-off value (maximizing sensitivity and specificity); the Youden Index was 0.42**(**Table 4; Fig. 2**)**. Importantly, a significant difference in AUC was observed between these two groups (*P* < 0.05), with TyG demonstrating diagnostic value exclusively in participants without overweight/obesity [AUC = 0.915,95%CI(0.88–0.95)], but not in those with BMI ≥ 24 kg/m² [AUC = 0.713,95CI(0.56–0.88)].

**Table 4:** Sensitivity, specificity, Youden index, Cut-off points, and AUC (95% CI) for predicting the risk of abnormal blood glucose metabolism events in populations with different types of reduced muscle mass

	Characteristic	Cut-off Point	AUC (95%CI)	Sensitivity	False Positive Rate (1- Specificity)	Youden Index^[1]^
TyG	Total	8.77	0.906 (0.56–0.88)	0.806	0.15	0.66
	BMI < 24 kg/m^2^	8.57	0.915 (0.88–0.95)	0.70	0.30	0.40
	BMI ≥ 24 kg/m^2^	8.88	0.713 (0.56–0.88)	0.77	0.35	0.42

Data are presented as Cut-off point, Area Under Curve,95% confidence intervals, Sensitivity,1-Specificity and Youden Index.

*OR* odd ratio, *TyG* triglyceride glucose index, *CI* confidence intervals, *AUC* Area Under Curve

[1]Fluss R, Faraggi D, Reiser B. Estimation of the Youden Index and its associated cutoff point. Biom J. 2005 Aug;47(4):458 − 72

### Subgroup analysis

Subgroup-specific odds ratios (ORs) with 95% confidence intervals (CIs) were reported to illustrate the magnitude and direction of associations within different strata.

**Fig. 3:**
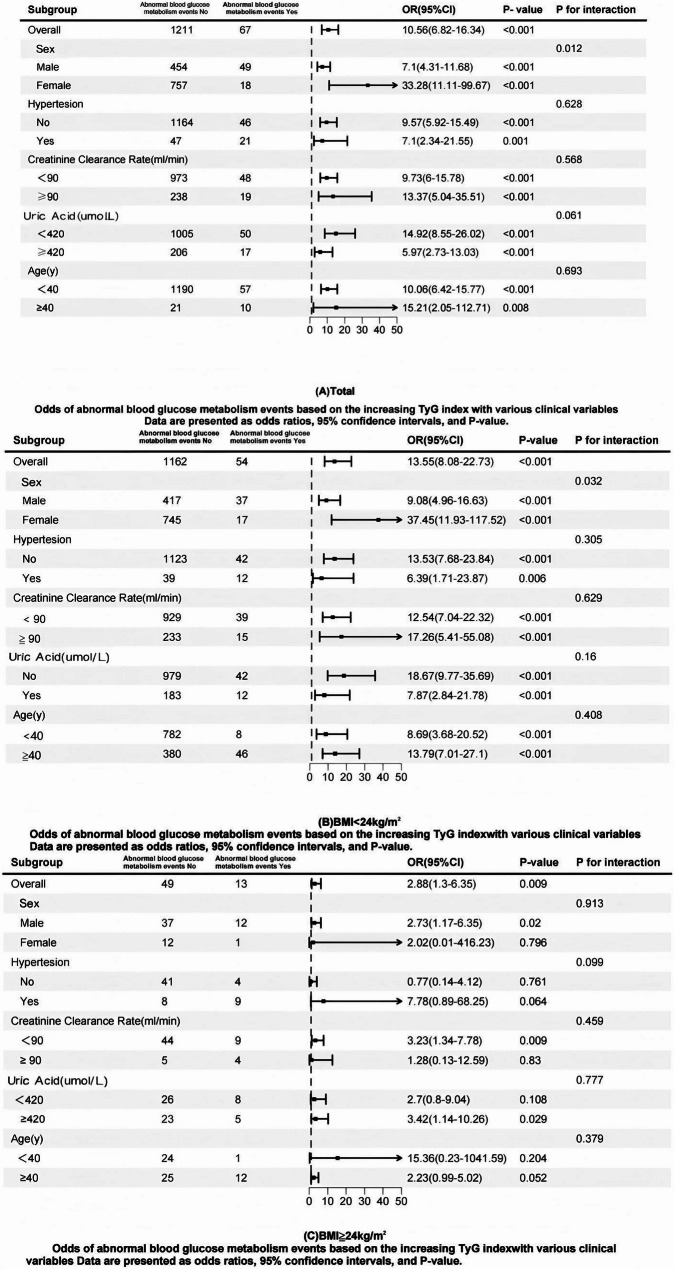
The Forest plot of Subgroup analysis and interaction analysis

The results showed a significant interaction effect between TyG levels and sex, indicating that the association between TyG and diabetes risk was stronger in women compared to men. No significant interaction effects were observed for other subgroup factors, such as age, hypertension, or creatinine clearance. These findings suggest that sex-specific factors may influence the utility of TyG as a predictive marker for diabetes risk**(**Fig. 3**)**.

## Discussion

The global prevalence of T2DM, representing 90% of diabetes cases, continues to rise, with IR in skeletal muscle and liver being a key pathogenic factor [24, 25]. While T2DM and sarcopenia exhibit bidirectional progression through shared mechanisms involving IR [26], the precise pathways remain incompletely understood. Our study provides new insights into this relationship by evaluating the TyG index as a predictor of dysglycemia in sarcopenic individuals.

Through multivariate-adjusted analyses, we found the TyG index consistently predicted abnormal glucose metabolism in subjects with reduced muscle mass, independent of obesity status **(**Tables 2 and 3; Fig. 3**)**. ROC curve analysis confirmed its predictive value (AUC > 0.5) across all sarcopenic subgroups **(**Table 4; Fig. 2**)**, supporting previous findings on lipid metabolism and IR [27–31]. Notably, the TyG index showed superior predictive performance in non-obese sarcopenic individuals compared to their obese counterparts.

This differential performance suggests distinct IR mechanisms may operate in sarcopenic populations with versus without obesity. While the TyG index appears particularly useful for detecting IR-related dysglycemia in non-obese sarcopenia, our findings imply that additional biomarkers may be needed for obese sarcopenic individuals. These results have important clinical implications for risk stratification and early intervention in different sarcopenic phenotypes.

Our findings align with prior research demonstrating the TyG index as a robust predictor of insulin resistance (IR) and dysglycemia in general populations [14, 16]. For instance, Guerrero-Romero validated the TyG index against hyperinsulinemic-euglycemic clamps (gold standard for IR assessment), reporting an AUC of 0.858 for diabetes prediction [14], which closely mirrors our results in sarcopenic individuals (AUC = 0.906). However, our research is consistent with the findings of Lou’s team [16], as we found that the TyG index demonstrates greater effectiveness in predicting diabetes or insulin resistance among Chinese patients with lower BMI values.

The differential predictive power of TyG in obese versus non-obese sarcopenic individuals likely reflects distinct pathophysiological pathways:

1. In non-obese sarcopenia: Muscle atrophy directly impairs insulin-mediated glucose uptake, increasing the reliance on lipid metabolism. The TyG index, which integrates triglycerides and glucose, thus becomes a sensitive marker of insulin resistance (IR) and impaired skeletal muscle glucose utilization [3, 27].
2. In sarcopenic obesity: Adipose tissue dysfunction (such as chronic inflammation and leptin resistance) may be the dominant mechanism of IR [6, 26], reducing the specificity of TyG.Asian populations have a higher risk of IR at lower body mass indices (BMIs), which may enhance the predictive value of TyG in our cohort compared to Western studies [9]. The TyG index offers a cost-effective tool for early dysglycemia screening in non-obese sarcopenic individuals, a population often overlooked in diabetes prevention strategies. For obese sarcopenia, combining TyG with adiposity markers (e.g., visceral fat index) may improve risk stratification [30].

### Limitations

This study exhibits several limitations. First, the cross-sectional design limits the ability to establish definitive causal relationships and excludes the potential for TyG to be dynamically linked with the development of abnormal blood glucose metabolism events. As a cross-sectional study, the outcome and the exposure were identified simultaneously, with the chronological order of events remaining ambiguous. Secondly, our findings pertain specifically to the Chinese population and may possess constraints in their applicability to a variety of racial groups. The limited sample size of individuals exhibiting reduced muscle mass alongside overweight or obesity in this study may lead to skewed results. Thirdly, there is an imbalance in the distribution of individual BMIs in this study. This can be attributed to the limited representation of participants with reduced muscle mass combined with obesity in the original data collected. The incidence of sarcopenic obesity is significantly lower than that of sarcopenia alone, potentially introducing bias into the results. We conducted a subsequent analysis to address concerns regarding excessively large confidence intervals for subgroups. To mitigate bias arising from uneven sample distribution, we employed Firth logistic regression. The results demonstrated that the Firth subgroup analysis confirmed the consistency of the statistical effect direction of TyG across all subgroups **(Supplementary Table 1)**. This finding reflects the true nature of the sample distribution. To address these limitations effectively, we are committed to continuously collecting and validating clinical data, collaborating with databases from other racial backgrounds for prospective studies, and conducting multi-database joint analyses to further explore and overcome these inherent limitations.This study investigates the association between sarcopenia and dysglycemic events, exploring the interplay of obesity and IR. The TyG index demonstrates superior predictive accuracy for abnormal glucose metabolism in non-obese sarcopenic individuals compared to their obese counterparts. These findings underscore distinct mechanistic pathways in sarcopenic obesity versus sarcopenia alone, offering novel insights for future research on targeted interventions.

## Conclusion

This study sought to evaluate the relationship between TyG and the risk of abnormal blood glucose metabolism events in individuals experiencing sarcopenia, while also comparing the detection capacity of this metric for the onset of abnormal blood glucose metabolism events in those with diminished muscle mass who does not concurrently exhibit overweight or obesity. The findings from our study demonstrate a positive correlation between the TyG index and abnormal blood glucose metabolism events onset in individuals with reduced muscle mass. Consequently, we advocate for the employment of the TyG index as the optimal predictive marker for tailored evaluation of abnormal blood glucose metabolism events risk in individuals who does not present with concurrent overweight or obesity, regardless of varying body mass indices.

## Supplementary Information

Supplementary Material 1: Supplementary Table 1. Firth logistic regression analysis of the association between TyG index and abnormal blood glucose metabolism events in populations with different types of reduced muscle mass.

## Data Availability

The datasets used and analyzed in the study are available upon reasonable request by the corresponding author.
