# Supplementary material for: Predictive effect of the triglyceride glucose index on abnormal blood glucose metabolism events in populations with sarcopenia: a cross-sectional study
Source: BMC Endocr Disord. 2025 Sep 30;25:212. doi: 10.1186/s12902-025-02026-8 (PMC12482197; doi:10.1186/s12902-025-02026-8)
Supplement: Supplementary file 1 — Supplementary Material 1: Supplementary Table 1. Firth logistic regression analysis of the association between TyG index and abnormal blood glucose metabolism events in populations with different types of reduced muscle mass. [file 12902_2025_2026_MOESM1_ESM.docx]

**Supplementary Material**

**Supplementary Table 1. Firth logistic regression analysis of the association between TyG index and abnormal blood glucose metabolism events in populations with different types of reduced muscle mass**

| **Variable** | **Count** | **Percent** | **beta** | **95%CI** | **P-value** |
| --- | --- | --- | --- | --- | --- |
| Overall | 1216 | 100 | 2.21 | 1.74，2.72 | <0.001 |
| Sex |  |  |  |  |  |
| Male | 454 | 37.3 | 1.84 | 1.36 ，2.38 | <0.001 |
| Female | 762 | 62.7 | 3.40 | 2.33 ，4.64 | <0.001 |
| Hypertension |  |  |  |  |  |
| No | 1165 | 95.8 | 2.31 | 1.79 ，2.89 | <0.001 |
| Yes | 51 | 4.2 | 1.61 | 0.74 ，2.78 | <0.001 |
| Creatinine Clearance rate（ml/min） |  |  |  |  |  |
| <90 | 968 | 79.6 | 2.18 | 1.63 ，2.78 | <0.001 |
| ≥90 | 248 | 20.4 | 2.27 | 1.37 ，3.31 | <0.001 |
| Uric acid（umol/L） |  |  |  |  |  |
| <420 | 1021 | 84 | 2.48 | 1.91，3.12 | <0.001 |
| ≥420 | 195 | 16 | 1.56 | 0.76 ，2.51 | <0.001 |
| Age（y） |  |  |  |  |  |
| <40 | 790 | 65 | 2.14 | 1.66 ，2.66 | <0.001 |
| ≥40 | 426 | 35 | 7.88 | 1.80 ，33.54 | <0.001 |

**Beta values, 95% confidence intervals (CI), and p-values are presented for overall participants and subgroups stratified by sex, hypertension status, creatinine clearance rate, uric acid levels, and age.**
